# Supplementary material for: The doctor knows or the evidence shows: An online survey experiment testing the effects of source trust, pro-vaccine evidence, and dual-processing in expert messages recommending child COVID-19 vaccination to parents
Source: PLoS One. 2023 Jul 21;18(7):e0288272. doi: 10.1371/journal.pone.0288272 (PMC10361505; doi:10.1371/journal.pone.0288272)
Supplement: S1 File — (DOCX) [file pone.0288272.s001.docx]

The doctor knows or the evidence shows: An online survey experiment testing the effects of source trust, pro-vaccine evidence, and dual-processing in expert messages recommending child COVID-19 vaccination to parents

Ava Irysa Kikut^1^*

^1^Annenberg School for Communication, University of Pennsylvania, Philadelphia, PA, USA

*Corresponding author

# Supporting Information

## S1: Treatment and Measures

**Treatment**

**Instructions:** On the following page, you will be given time to read a quote. After 30 seconds, an arrow will appear to take you to the next section. Please read the quote carefully. You will be asked questions about what you read.

**S1A Table. Recommendation message for each condition**

|  | No Trust Cue | Trust Cue |
| --- | --- | --- |
| No Evidence | “I recommend the COVID-19 vaccine to anyone who is eligible.” – Dr. Taylor Clark | “As a parent, I know conversations around the COVID-19 vaccine have been really complicated. Sometimes it can be hard to know what the right thing to do is. I want to do everything I can to protect my kids and to make sure they live the healthiest life. That is the most important thing to any parent. I tell parents I wouldn’t recommend the vaccine for their children if I didn’t think it was safe for my own family. I got my children vaccinated to keep them healthy. I recommend the COVID-19 vaccine to anyone who is eligible.” – Dr. Taylor Clark |
| Evidence | “The COVID-19 vaccine is well-researched. Clinical trials with thousands of children have shown the vaccine is over 91% effective. In the rare cases when vaccinated children are infected with COVID-19, they are protected from serious symptoms and long-term complications. Less than 0.01% of children have experienced serious side effects from the vaccine. Over 700 children have died from COVID-19 and no children have died from the vaccine. The data is clear. I recommend the COVID-19 vaccine to anyone who is eligible.” – Dr. Taylor Clark | “As a parent, I know conversations around the COVID-19 vaccine have been really complicated. I tell parents I wouldn’t recommend the vaccine if I didn’t think it was safe for my own family. I got my children vaccinated. Clinical trials have shown the vaccine is over 91% effective. Less than 0.01% of children have experienced serious side effects. Over 700 children have died from COVID-19 and none have died from the vaccine. The data is clear. I recommend the COVID-19 vaccine to anyone who is eligible.” – Dr. Taylor Clark |

## Measures

## Perceived message effectiveness

**Instructions:** The following questions ask you to provide your opinions about the quote. Please answer truthfully, there are no right or wrong answers. (*1=Strongly disagree; 2=Somewhat disagree; 3=Disagree; 4=Agree; 5=Somewhat agree; 6=Strongly agree*)

*[Statements presented in random order]*

- This quote will be effective in encouraging parents to get their children vaccinated against COVID-19.
- This quote increases my confidence about getting my child vaccinated against COVID-19.
- This quote makes me more concerned about getting my child vaccinated against COVID-19. *(reverse coded)*
- The statements in this quote make a strong case that COVID-19 vaccines are safe for children.
- This quote put thoughts in my head about the advantages of getting my child vaccinated against COVID-19.
- This quote is unlikely to convince a parent to get their child a COVID-19 vaccine. *(reverse coded)*

**Pro-Vaccine Behavioral beliefs**

**Instructions:** How much do you disagree or agree with the following statements about the COVID-19 vaccine that has been approved for children in the United States? Please answer truthfully, there are no right or wrong answers.  (*1=Strongly disagree; 2=Disagree; 3=Somewhat disagree; 4=Undecided; 5=Somewhat agree; 6=Agree; 7=Strongly agree*)

*[Statements presented in random order]*

- Getting the COVID-19 vaccine is a good way to protect my child against serious illness.
- Getting the COVID-19 vaccine benefits my child.
- The COVID-19 vaccine is effective at preventing COVID-19.
- The COVID-19 vaccine is unsafe for children. *(reverse coded)*
- Getting the COVID-19 vaccine is more dangerous for children than getting infected with COVID-19. *(reverse coded)*

**Perceived source trustworthiness**

**Instructions:** The following questions ask about your feelings toward Dr. Taylor Clark, the speaker of the quote. Please answer truthfully, there are no right or wrong answers. (*1=Strongly disagree; 2=Somewhat disagree; 3=Disagree; 4=Agree; 5=Somewhat agree; 6=Strongly agree*)

*[Statements presented in random order]*

- The speaker is untrustworthy. *(reverse coded)*
- The speaker does not care about my child. *(reverse coded)*
- I believe the speaker is sincere.
- The speaker cares about my child’s well-being.
- I trust the speaker to be honest.
- The speaker is sensitive to my concerns as a parent.

**Perceived evidence**

- The quote I read for this survey mentioned research that has been done on COVID-19 vaccines with children. (*1=True; 0=False*)
- The quote I read for this survey gave me lots of evidence in favor of getting my child vaccinated. (*1=Strongly disagree, 2=Disagree, 3=Agree, 4=Strongly agree*)

**Influence over whether child receives vaccine**

- For your youngest child, do you make the decision about the vaccinations your child receives? (*1=Yes, 2=No, 3=Decisions made jointly with another parent/guardian)*

**Unvaccinated child**

*[Randomly presented as either first or last question in survey]*

- Are you the parent or guardian of a child who is **5 to 17 years old** and has **not** received a COVID-19 vaccine? (*1=Yes, 2=No, 3=Don’t know*)

## S1B Table: Development of Perceived Message Effectiveness Scale from Zhao et al. (2011)

| Description of Item | Original Perceived Argument Strength Scale Item | Updated Item for Current Study | Justification for Changes |
| --- | --- | --- | --- |
| Assessment of message’s potential effectiveness for others with whom one relates | The statement would help my friends *[insert target behavior].* | This quote will be effective in encouraging parents to *get their children vaccinated against COVID-19*. | Modified to focus on other parents, as relevant relatable others |
| Assessment of message’s effect on one’s ability to do the target behavior | The statement helped me feel confident about how best to *[insert target behavior].* | This quote increases my confidence about *getting my child vaccinated against COVID-19.* | Slight wording change to better fit context |
| Reverse coded statement pertaining to the message decreasing one’s desire to engage in the target behavior | The statement put thoughts in my mind about not wanting to *[insert target behavior].* | This quote makes me more concerned about *getting my child vaccinated against COVID-19.* | Slight wording change to better fit context |
| Assessment of message’s argument strength | Is the reason the statement gave for *[insert target behavior]* a strong or weak reason? | The statements in this quote make a strong case that *COVID-19 vaccines are safe for children*. | Converted to a statement that works with other battery items in a single Likert scale |
| Assessment of message’s effect on whether one thinks about target behavior | The statement put thoughts in my mind about wanting to *[insert target behavior].* | This quote put thoughts in my head about *the advantages of getting my child vaccinated against COVID-19.* | Slight wording change to better fit context |
| Assessment of whether message is convincing in general | The statement is a reason for *[insert target behavior]* that is convincing. | This quote is unlikely to convince a parent to get their child a COVID-19 vaccine. | Wording change to better fit context |
| Assessment of overall agreement with the message | Overall, how much do you agree or disagree with the statement? | *Excluded* | Too similar to behavioral belief measurement. |
| Assessment of message believability | This statement is a reason for *[insert target behavior]* that is believable. | *Excluded* | Too similar to behavioral belief measurement. |

*Note.* This table illustrates each item recommended in the perceived argument strength scale provided by Zhao et al. (2011) and the corresponding item adapted for the perceived message effectiveness (PME) scale used in the current study. As described in the final column of the table, modifications were made to fit the population, behavior of interest, and experimental context. Principal components analysis on the modified scale showed the eigenvalue for the first component (“This quote will be effective in encouraging parents to get their children vaccinated against COVID-19”) was 3.75 and no other component had an eigenvalue meaningfully higher than 1. All other components loaded positively on the first component. Eigenvector loadings were 0.458, 0.464, 0.210, 0.457, 0.474, and 0.314.
