# Supplementary material for: The doctor knows or the evidence shows: An online survey experiment testing the effects of source trust, pro-vaccine evidence, and dual-processing in expert messages recommending child COVID-19 vaccination to parents
Source: PLoS One. 2023 Jul 21;18(7):e0288272. doi: 10.1371/journal.pone.0288272 (PMC10361505; doi:10.1371/journal.pone.0288272)
Supplement: S2 File — (ZIP) [file pone.0288272.s002.zip › S2.AKikutPlosONE2023_Codebook.docx]

The doctor knows or the evidence shows: An online survey experiment testing the effects of source trust, pro-vaccine evidence, and dual-processing in expert messages recommending child COVID-19 vaccination to parents

Ava Irysa Kikut^1^*

^1^Annenberg School for Communication, University of Pennsylvania, Philadelphia, PA, USA

*Corresponding author

# Supporting Information

**S2: Data Codebook**

**S2A Table. Variable names and coding**

| Variable name | Stata file name | Description/values |
| --- | --- | --- |
| Trust cue | anyparent | 1=trust cue  0=no trust cue |
| Evidence | anyevidence | 1=evidence  0=no evidence |
| Trust cue only | parent | 1=trust-cue only condition  0=all other conditions |
| Evidence only | evidence | 1=evidence only condition  0=all other conditions |
| Trust cue + evidence | parentevidence | 1=trust-cue + evidence condition  0=all other conditions |
| Control | control | 1= control condition  0=all other conditions |
| PME score | pme | Perceived message effectiveness scale |
| PME items | Q26_1-6  Q36_1-6  Q46_1-6  Q56_1-6 | Perceived message effectiveness items (1 and 2 recoded); *Q26=respondents for evidence condition, Q36=responses for trust+evidence, Q46=responses for trust only, Q56=responses for control* |
| Belief score | belief | Pro-vaccine belief scale |
| Belief items | b1-5 | Behavioral beliefs (4 and 5 recoded) |
| Trust score | trust | Perceived trustworthiness scale |
| Trust items | Q24_1-6  Q34_1-6  Q44_1-6  Q54_1-6 | Perceived trustworthiness items (3 and 6 recoded)  *Q24=respondents for evidence condition, Q34=responses for trust+evidence, Q44=responses for trust only, Q54=responses for control* |
| Perceived evidence score | evman | Perceived evidence |
| Evidence item | Q61 & Q62 | Perceived evidence questions |
| Decision-making parent | decisionmaker | 1=yes; 2=no; 3=decisions made jointly |
| Unvaccinated children | unvax | 1=any eligible unvaccinated child; 0=no eligible unvaccinated child (combines responses from Q64 and Q64b) |
| Unvaccinated children (pre) | Q64 | Any child unvaccinated question asked pre-survey |
| Unvaccinated children (post) | Q64b | Any child unvaccinated question asked post-survey |
| Completed survey | Finished | 1=Yes  0=No |

*Note.* Complimentary Stata file: S2.AKikutPlosONE2023_RawData.dta
