# Supplementary material for: The doctor knows or the evidence shows: An online survey experiment testing the effects of source trust, pro-vaccine evidence, and dual-processing in expert messages recommending child COVID-19 vaccination to parents
Source: PLoS One. 2023 Jul 21;18(7):e0288272. doi: 10.1371/journal.pone.0288272 (PMC10361505; doi:10.1371/journal.pone.0288272)
Supplement: Attachment — Submitted filename: Review of PONE-D-22-10305.pdf [file pone.0288272.s003.pdf]

This manuscript reports on the results of an online study which employs an experimental design to test whether the presentation of COVID-19 vaccine information to parents, including source trustworthiness and evidence of vaccine efficacy, differentially and interactively impact self-reported intentions to vaccinate their child and belief regarding vaccine safety and efficacy for children. The topic is highly relevant, and the study design is novel. Additionally, the author is commended for pre-registering their hypotheses prior to data collection. However, there are significant concerns throughout the manuscript that need to be addressed. Briefly, these include a lack of appropriate scientific language, inadequate statistical analyses, and a general lack of detail regarding crucial aspects of the sample and methodology.

OVERALL/WRITING

1. Several grammatical errors were identified throughout the manuscript (e.g., lines 34-35). Please revise carefully to ensure it is free from such errors.
2. I would encourage the author to review style guides for scientific writing. The manuscript would be greatly improved if for example instances of first person "I" were removed, and rhetorical questions were changed to statements (especially in the introduction).
3. There is a lack of consistent language when referring to specific constructs throughout the manuscript. For example, the author refers to the interaction between peripheral and central cues on line 108, but earlier describes them as type 1 and type 2 processing.
4. When referring to provide evidence on vaccine efficacy/safety, I would encourage the author to explicitly state this at very mention even if it's "vaccine evidence" just to make it clear for the reader.

ABSTRACT

1. Please consider revising the hypothesis and design section of the abstract to improve clarify. For example, presenting to the reader that you are interested in the effects of x on x descriptively, rather than stating that you are interested in the main effects and interactions (line 17). Additionally, referring to aspects of the design in an abbreviated manner (e.g., "trust cue vs. no trust cue" on line 19) can be confusing as this has not yet been explained to the reader.
2. Please use the term *significant* when describing a main effect or interaction (if appropriate) as simply stating that there was a main effect or interaction does not provide adequate detail to the reader.

INTRODUCTION

1. Some of the writing in the introduction is unclear or lacks detail. For example, on line 42, the author states "how do the effects of source trust differ" but does not name the outcome variable only the moderator (present of evidence). On line 94, the author does not specify the directionality of the relationship named. Please revise the entire manuscript to ensure that statements regarding the relationships among variables or constructs are complete and clear to the reader.
2. On lines 66-76, the author describes the construct of source credibility. Overall, this section is a bit confusing to the reader (stating that credibility can be broken into trustworthiness and perceived expertise and that trustworthiness can be further broke into

trustworthiness and goodwill). Finally, there is the addition of including measures of honestly and empathy at the end. I would encourage the author to make this section much more concise and describe only what is necessary to describe what is measured. Additionally, more rationale should be given for the inclusion and purpose of honestly and empathy.

METHODS

1. The Forthright Capabilities panel should be explained in detail. Is this a labor market platform similar to MTurk? How are participants recruited? How many are in the pool? How was this study advertised to them? Also, the author mentioned that the demographic data matches the US population but does not provide any of this data. Please include descriptive statistics for age, gender, sex, ethnicity, race, education, etc. as available. These are crucial aspects to consider given the nature of this study and potential application to different groups within the US.
2. The author mentioned on line 136 that to increase statistical power, the two samples were combined. Was a power analysis conducted? If not, how does the author justify the sample size?
3. On line 135, the author mentions that the survey was conducted through an academic course but does not provide further detail. Please explain how participants were recruited and how they qualified within the course. Also, was this a course at a given university or other institution?
4. In the Design section, the author names the conditions (e.g., "trust cue vs. no trust cue") prior to explaining what they are. To provide clarity to the reader, please describe the conditions first, then the analytic design second.
5. Lines 140 to 162 lack adequate detail and clarity regarding the procedure. Please revise to guide the reader step-by-step through how participants started the experiment survey, including where they were, what survey software was used, etc. A figure would help clarify the design of this study to clearly identify each step of the procedure and where the conditions branched off. Regarding lack of detail, the author should provide samples of the materials presented (e.g., naming the specific measures on line 146, including short quotes of each cue type, etc.).
6. The author states that the trust + evidence condition utilized a trimmed down cue. How was it trimmed down? The author should mention why this decision was made (beyond keeping the time the same) in the discussion, especially given that changing this cue could potentially introduce error or reduce the ability to truly compare conditions.
7. On line 164, the author mentions that the manipulation check "ensured" – please revise as manipulation checks are only limited post-hoc examinations that estimate whether the manipulation *may* have had the intended effect.
8. The method of describing different measures in the methods section is a bit confusing to the reader. I would recommend breaking it down with subheadings for each measure (in the order they were administered) and then describing the order of administration in the earlier design section. Please provide psychometric information as available for each measure (e.g., internal consistency, test re-test reliability, etc.) and whether it has been validated previously. Additionally, please provide an example item for each measure in quotes.

9. The author mentions that the two measures regarding child vacation status and control over vaccine choice were split (50% at the beginning, 50% at the end). How could this have differentially impacted groups? The author should re-run the analyses controlling for this as a variable to rule out the introduction of error.
10. The analytic plan section should be expanded with more detail (e.g., what software was used?). The author should also describe the plan for making pairwise comparison following each main effect or interaction.

RESULTS

1. On line 213, the author mentions "65% of participants" but is this out of the total 398? If so, would this bring the final sample to 258? Please clarify.
2. For tables 1-2, there is no need to indicate what M and SE stand for as it can be assumed the reader knows. Additionally, there is no need to state that the measure contained six items from 1-6.
3. On line 223, the author states that all manipulation conditions had higher means than the control, but was this a significant difference? If not, this may mislead the reader. Please clarify.
4. When describing main effects and interactions (e.g., line 234), please use the term *significant* to clarify.
5. When reporting results from ANOVAs throughout the results section, please provide the F value, degrees of freedom, confidence interval, and effect size.
6. It does not appear that the author conducted pairwise comparisons (if so, this is unclear) but merely provided a descriptive comparison of means. As it stands, comparing means without any type of test is not a statistically valid way to make conclusions from data. Please clarify.
7. As mentioned previously, please include effect sizes for every statistical test run.
8. At several points, the author mentions that there are results that do not reach "traditional significance." This is a massive concern as it implies to the reader that the effect was there but simply didn't meet a certain threshold. This is not the case as any effect that does not reach significance must be attributed as no different than random. Please remove all instances of this phrase throughout the results and discussion and use explicit language to identify that in these cases, there was <u>no</u> effect found.

DISCUSSION

1. Please revise the limitations section to include all of the aforementioned concerns in this review that cannot be addressed.
